# An Incidental Finding of Asymptomatic Gastric Diverticula

**DOI:** 10.7759/cureus.31250

**Published:** 2022-11-08

**Authors:** Hussain A Al Ghadeer, Ali S Al Hassan, Nouh H AlAli, Abdullah J Almusharaf, Mishael M AlDawood, Manal M Alghazal, Sajidah M Almeshal, Fatimah S Albattat

**Affiliations:** 1 Pediatrics, Maternity and Children Hospital, AlAhsa, SAU; 2 Gastroenterology and Hepatology, Maternity and Children Hospital, AlAhsa, SAU

**Keywords:** alahsa, saudi arabia, endoscopy, foreign body, congenital, gastric diverticulum

## Abstract

Gastric diverticula (GD) are the rarest of the gastrointestinal diverticula and are characterized by protrusions of the stomach wall, that can either be congenital or acquired. Despite the fact that the majority of GD are asymptomatic and are detected inadvertently during endoscopy or gastrointestinal (GI) series studies, they might present with a variety of symptoms, including abdominal pain, vomiting, and weight loss. In mild symptomatic instances, GD is treated conservatively with antacids, but surgical excision is indicated for refractory gastric diverticula with persistent symptoms or complications. We represent an incidental finding of asymptomatic gastric diverticulum through endoscopy for a 12-year-old Saudi male who presented after foreign body ingestion.

## Introduction

Gastric diverticula are rare in children, with only around 4% of GD reported in patients less than 20 years old. It affects both genders equally and occurs most often in the fifth and sixth decades of life. The prevalence of GD has been estimated to vary from 0.01% to 0.11% in upper gastrointestinal endoscopies and up to 0.02% in autopsy studies [1,2]. Diverticula are divided into two types: congenital and acquired. Congenital conditions affecting all gastrointestinal layers made up the vast majority of the cases. Typically, congenital GD is found 2-3 cm below the gastroesophageal junction, at the dorsal wall of the fundus. The severity of the symptoms determines how to treat gastric diverticula. For incidental findings and asymptomatic patients, conservative therapy is advised. On the other hand, complicated cases, including ulceration, hemorrhage, etc., require surgical intervention for resection [3].

## Case presentation

After an hour of ingestion of a foreign body, a 12-year-old Saudi male was referred to our institution at Maternity and Children Hospital, AlAhsa, Eastern Region, Saudi Arabia. The patient was stable and asymptomatic at the time of presentation. The foreign body was metallic, in the form of a key. He denied experiencing dyspnea, dysphagia, coughing, nausea, vomiting, abdominal discomfort, melena, or hematemesis. His appetite and bowel motions were normal. He had no history of substance abuse, smoking, weight loss, or gastrointestinal tract diseases. There is no evidence of an inherited disease in the family.

Physical examination revealed that he was conscious, alert, and not in distress and that his vital signs were within normal limits. His height and weight were both in the 50th percentile. Local examination revealed a non-distended, soft, loose abdomen with no organomegaly and no evidence of guarding or tenderness, with intact bowel sounds. The results of the other systemic examinations were normal.

**Figure 1:**
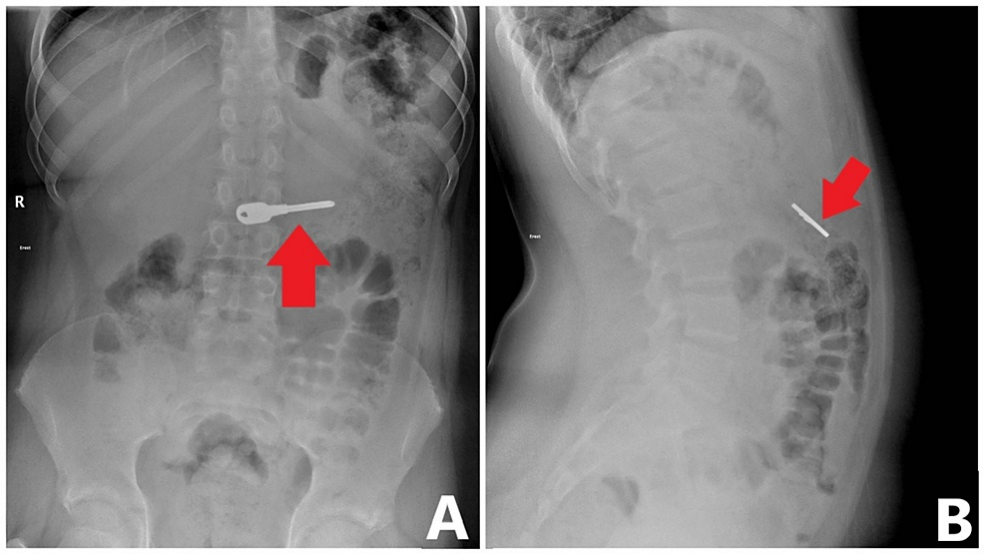
Erect abdominal X-rays reveal a radiopaque, metallic foreign body (red arrows)

Laboratory results, including a complete blood count, coagulation profile, liver and renal function tests, and urine analysis, were all within normal limits. Erect abdominal X-rays (Figures 1A, 1B) revealed a radiopaque metallic foreign body (key) in the stomach.

**Figure 2:**
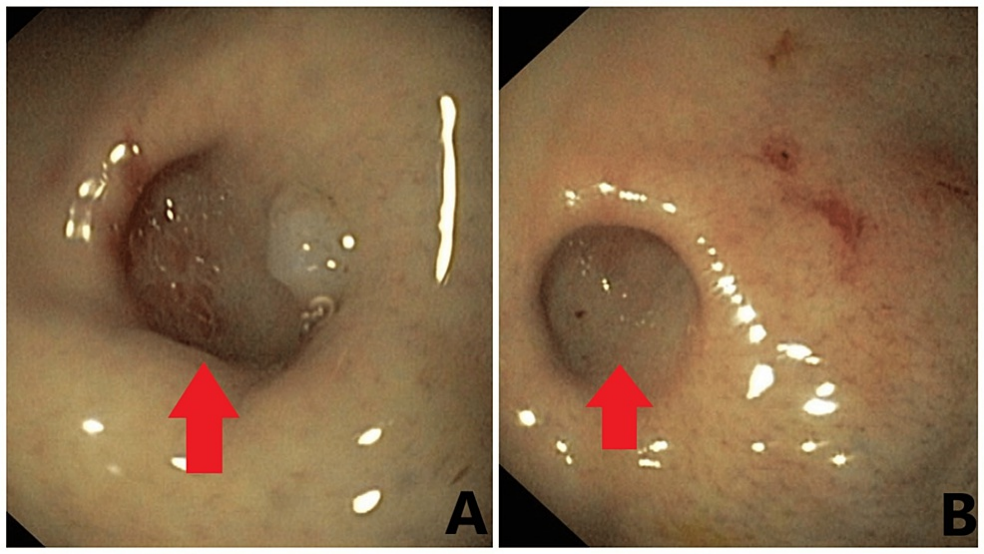
Endoscopic view of the gastric diverticulum (red arrows) located in the body of the stomach Size of the gastric diverticulum: 20*20*10 mm

At the time, an esophagogastroduodenoscopy (EGD) was undertaken, and the key was successfully removed without complications. Furthermore, an accidental discovery of diverticulum was visible, surrounding the pylorus and gastric body with an estimated size of 20*20*10 mm (Figures 2A, 2B) with normal seeming mucosa and no other pathologies of the esophagus, stomach, or duodenum detected during endoscopy.

Because the patient was asymptomatic, no medical or surgical intervention was performed.

## Discussion

GD are the rarest among the diverticula seen in other parts of the gastrointestinal system. Only 4% of individuals less than 20 years old have gastric diverticula, which typically appear between 20 and 60 years old. Prevalence estimates vary from 0.04% (165/380,000) in upper GI contrast radiography studies to 0.01-0.11% in upper GI endoscopies to 0.02% (6/29,900) in autopsy investigations [1,4-6]

Congenital (true) and acquired (false) diverticula are the two forms of GD. True diverticula include all layers of the stomach wall. False diverticula, on the other hand, don't. Furthermore, false diverticula are subdivided into pulsion and traction. Pulsion diverticula form as a consequence of increased intraluminal pressure caused by prolonged coughing, obesity, or pregnancy. Traction diverticula, on the other hand, form as a result of contractile forces caused by an adjacent inflammatory process or perigastric adhesions caused by concurrent diseases such as peptic ulcer disease, pancreatitis, cholecystitis, cancer, gastric outlet obstruction, and gastroesophageal reflux disease [3]. Congenital diverticula, which account for 70-75% of all GD, are situated mostly on the posterior part of the gastric cardia or fundus, while acquired diverticula are frequently found within the gastric antrum [7]. In this report, GD was most likely to be congenital with no supportive evidence of acquired GD. Also, it was confined to the gastric body.

Although most cases of GD are asymptomatic and discovered accidentally, patients may exhibit non-specific symptoms, such as abdominal pain, emesis, weight loss, and iron deficiency anemia, or consequences like bleeding, gastroesophageal reflux, and perforation [4,8]. Because of this, the optimal diagnostic approach remains uncertain. Upper gastrointestinal series (UGI) with contrast is widely used as a diagnostic tool for GD. Nevertheless, a large review found that around 5% of GD are missed by UGI [2]. As a result, EGD remains the gold standard for diagnosing GD [1]. Our patient was asymptomatic and diagnosed by EGD as a secondary outcome after the removal of the foreign body.

The treatment of GD is mostly determined by the severity of the presenting complaints as well as the extent of the diverticulum. However, conservative therapy with proton pump inhibitors, histamine (H2) receptor antagonists, or antacids may give short-term symptom alleviation. In contrast, situations that are symptomatic and/or complex, such as ulceration, bleeding, perforation, and obstruction, should be excised [1,7]. GD larger than 4 cm in diameter are more likely to exhibit complications [9,10]. Because our patient was asymptomatic, no medical treatment was started.

## Conclusions

The symptoms of GD might vary, even though the majority of cases are asymptomatic and are found by chance during standard diagnostic testing. The occurrence of life-threatening complications necessitates surgical intervention.
